# Synergistic effects of the components of global change: Increased vegetation dynamics in open, forest-steppe grasslands driven by wildfires and year-to-year precipitation differences

**DOI:** 10.1371/journal.pone.0188260

**Published:** 2017-11-17

**Authors:** Miklós Kertész, Réka Aszalós, Attila Lengyel, Gábor Ónodi

**Affiliations:** 1 Institute of Ecology and Botany, MTA Centre for Ecological Research, Vácrátót, Hungary; 2 MTA Centre for Ecological Research, GINOP Sustainable Ecosystems Group, Tihany, Hungary; Ilam University, ISLAMIC REPUBLIC OF IRAN

## Abstract

Climate change and land use change are two major elements of human-induced global environmental change. In temperate grasslands and woodlands, increasing frequency of extreme weather events like droughts and increasing severity of wildfires has altered the structure and dynamics of vegetation. In this paper, we studied the impact of wildfires and the year-to-year differences in precipitation on species composition changes in semi-arid grasslands of a forest-steppe complex ecosystem which has been partially disturbed by wildfires. Particularly, we investigated both how long-term compositional dissimilarity changes and species richness are affected by year-to-year precipitation differences on burnt and unburnt areas. Study sites were located in central Hungary, in protected areas characterized by partially-burnt, juniper-poplar forest-steppe complexes of high biodiversity. Data were used from two long-term monitoring sites in the Kiskunság National Park, both characterized by the same habitat complex. We investigated the variation in species composition as a function of time using distance decay methodology. In each sampling area, compositional dissimilarity increased with the time elapsed between the sampling events, and species richness differences increased with increasing precipitation differences between consecutive years. We found that both the long-term compositional dissimilarity, and the year-to-year changes in species richness were higher in the burnt areas than in the unburnt ones. The long-term compositional dissimilarities were mostly caused by perennial species, while the year-to-year changes of species richness were driven by annual and biennial species. As the effect of the year-to-year variation in precipitation was more pronounced in the burnt areas, we conclude that canopy removal by wildfires and extreme inter-annual variability of precipitation, two components of global environmental change, act in a synergistic way. They enhance the effect of one another, resulting in greater long-term and year-to-year changes in the composition of grasslands.

## Introduction

In recent years, human-induced global change and its effects on ecosystems have been one of the most important research topics in ecology [1,2]. As an element of global change, climate change has major influence on grasslands and grassland-woodland complexes [3,4], altering their extent, species richness and composition [5,6]. The ecological impacts of climate change components, i.e. the rise of temperature and changes in precipitation, are the subject of intensive research [7,8]. Among other variables, the distribution of precipitation is one of the most important regulating factors of ecosystems, especially in arid and semiarid grasslands [9,10]. The year-to-year variation in precipitation can be a dominant driver of species turnover in arid or semiarid ecosystems [11] because both previous and current year precipitation amounts are important regulating factors of species diversity and composition in these communities [12]. However, few studies have investigated the relationship between year-to-year species dynamics and year-to-year differences in precipitation [13].

Land use change is another major component of global change. Introduction and abandonment of crop production, changes in grazing pressure, deforestation and afforestation are key components of this transformation [14]. In the temperate climate zone, semi-arid grasslands and woodlands have become more fire-prone in the last decades as an indirect effect of the land use change and climate change [15–17]. Even in habitats where fire is part of the natural disturbance regime, its increasing frequency and severity change the composition and pattern of the mosaic of grassland and woody vegetation [18]. Increasing fire frequency may lead to a decrease in shrub or wood cover [19]. The effects of fire are studied from several perspectives, such as resilience of communities to fire, post-fire succession processes, regeneration, or fire as a management option [20]. The removal of the woody canopy of wood-grassland habitat complexes makes the microclimate warmer [21] and alters the species composition and richness of the herbaceous layer [22].

In densely-populated areas of Central and Southern Europe, less productive areas have been left in a semi-natural state, thus, they often serve as biodiversity hotspots in depleted agricultural landscapes. In Hungary, the forest-steppe on sandy soil characterized by juniper and poplar is a grassland-woodland vegetation mosaic [23], which is relatively less-altered. This habitat type has high conservation value since it harbors many unique communities and species, including several that are endemic [24]. In the last decades, as a consequence of extensive afforestation of the highly combustible black pine [25], about 50% of the juniper-poplar forest-steppe had been burnt by wildfires (Kiskunság National Park, unpublished). The increased number and extent of wildfires have transformed the forest-steppe vegetation to a grassland habitat. Our previous results showed that in this community, changes in patch types of the grassland vegetation are more frequent in the fire-made gaps, independent of the time since fire [26]. We also found that these changes were affected by the amount of precipitation on the burnt areas. As the year-to year differences are predicted to be more extreme in the future [27], this raises the question of how precipitation contributes to year-to-year species-level vegetation dynamics in burnt and unburnt grasslands.

In this paper, we report on our study of the impacts of wildfires on the long-term changes in species composition in grasslands of a forest-steppe ecosystem. We aimed to answer two questions regarding long-term as well as year-to-year vegetation changes and their differences in burnt and unburnt areas. 1) Does compositional dissimilarity increase with time, and if so, is this increase different in burnt and unburnt areas? 2) Do year-to-year changes in species richness depend on year-to-year differences in precipitation, and if so, is this dependence different in burnt and unburnt areas? We studied the above questions in relations to all vascular plant species, as well as separately for short-lived (i.e. annual and biennial) and long-lived (perennial herb and woody) plants. We investigated two discrete sites characterized by the same habitat complex.

## Materials and methods

### Study sites

Study sites were located in the Kiskunság National Park, in the Bugac (N 46° 39.0’, E 19° 36.4’) and Orgovány (N 46° 47.7’, E 19° 27.3’) sites of the KISKUN LTER project [28], central Hungary. Kiskunság Nemzeti Park (Kiskunság National Park) provided permission to carry on field sampling in protected natural areas. The distance between the study sites is 20 km. Elevation of the study area ranges from 115 to 125 meters in the Bugac site, and from 112 to 121 meters in the Orgovány site. The climate is temperate continental with sub-Mediterranean effect [29]. The mean annual precipitation is 500–550 mm, and monthly mean temperatures range between -1.8°C (January) and 21°C (July). The soil is calcaric arenosol, formed of coarse sand with high calcium carbonate (5–10%) and low (<1%) humus content [30]. Study areas have been protected since 1975 as core areas of the national park, so no agricultural activity has occurred recently. The main historical human disturbances were extensive grazing by cattle and sheep until the mid-19^th^ century, deforestation of some areas for military purpose during the 19^th^ and 20^th^ centuries, and afforestation by alien tree species since the 1950’s [31,32]. The study sites are at the western edge of the Eurasian forest-steppe zone. The potential vegetation is an edaphic mosaic of open sand grasslands and woodlands [23,30]. Grasslands are co-dominated by perennial grasses: *Festuca vaginata* Waldst. & Kit. ex Willd., *Stipa pennata* L. Woody vegetation is dominated by juniper (*Juniperus communis* L.), and poplar species (*Populus alba* L., and *Populus nigra* L.), forming a shrub cover typically three to six meters high. The woody canopy is often sparse due to historical human disturbances, but can reach a continuous matrix up to approximately 75% cover, as occurred in our study sites. Thus, in the unburnt areas the grasslands are fragmented into patches, typically between 50 and 500 square meters. While wildfire events differ in their severity, in this vegetation complex fire always exterminates junipers in the burn areas, usually decreases the cover of the woodland matrix to a maximum of 10%, and induces the re-sprouting of poplar species. During the secondary succession process, grassland species and sprouts of poplar species occupy the burnt areas [26,33].

### Experimental design and data collection

We compared vegetation composition changes in open sand grassland patches of unburnt forest-steppe vegetation and grassland patches of burnt forest-steppe. The latter patches were formerly isolated from each other by juniper and poplar woody vegetation. At the beginning of the study, 21 years after the fire in Bugac, and 2 years after the fire in Orgovány (see Table 1), we chose grassland patches (ten patches per hectare [26]) in both sites to cover the compositional variation of the grasslands. Although we sampled grassland patches, sprouts of woody poplar species and young specimens of introduced alien black pine (*Pinus nigra* F.J.Arnold) also occurred in the sample.

**Table 1:** Summary of observation areas.

Site	Year of fire	Sampled years	Area	Patch number	Quadrat number
Orgovány	2000	2002–2013	Burnt	20	5 x 20
			Unburnt	20	5 x 20
Bugac	1976	1997–2011	Burnt	10	5 x 10
			Unburnt	10	5 x 10

Results from initial years in these study sites were analyzed in Ónodi et al. [26], and the present research includes the results from additional years (Table 1). In the case of the Orgovány site, which was partially burnt in 2000, 20 grassland patches were selected in both burnt and unburnt areas. They were sampled annually from 2002 until 2013, providing a chronosequence of 2×20 patches for 12 years. For the Bugac site, which was partially burnt in 1976, we selected 10 patches in burnt areas and 10 patches in unburnt areas, and sampled them annually since 1997. We analyze the Bugac dataset until 2011, because another large wildfire changed the vegetation in 2012. Thus for the Bugac site we used a chronosequence of 2×10 patches for a period of 15 years.

In each patch, five permanent 1×1 m^2^ quadrats were sampled [26]. In order to track seasonal variation in species composition during the vegetation period, occurrence of vascular plant species, including annuals, biennials, perennial herbs and woody species, was recorded in the sampling quadrats twice a year (in late May to early June and in late September to early October). Presence-absence data were obtained by combining spring and fall recordings in all five quadrats, which resulted in a single record for each patch for each year. Precipitation data were collected by regional meteorological stations in Bugac and Fülöpháza (6 km from the Orgovány site).

### Statistical analyses

We investigated the variation in species composition as a function of time using distance decay methodology [34]. In community ecology and biogeography, distance decay models are generally used to calculate pairwise dissimilarities of communities in terms of their spatial, temporal or environmental distances, thus both the dependent and the explanatory variables are in form of distance matrices. When analyzing temporal gradients, year-to-year variation in species composition without directional changes can be detected in the form of non-significant relationships between dissimilarity and distance, i.e. time lag (or time interval). By contrast, directional long-term compositional change results in a statistically significant slope parameter of the linear function between dissimilarity and time lag.

Several indices are available for expressing community patterns with pairwise dissimilarities [35]. We used two indices, one of them sensitive to compositional turnover and one expressing change in species richness.

For expressing compositional dissimilarity, we used the complement of the simple matching coefficient SM:

$$1-SM=1-\frac{number\;of\;common\;presence\;of\;species+number\;of\;common\;absence\;of\;species}{total\;number\;of\;species}$$

The total number of species was determined separately for the two sites (see Table 1). Then 1-SM between pairs of samples of different years on the same grassland patch was calculated. Next, dissimilarities representing the same pair of years were averaged across grassland patches, separately for the burnt and the unburnt grasslands. These average dissimilarities were used as a response matrix in the distance decay model. 1-SM is a symmetric measure of dissimilarity and it can get only non-negative values.

The second index expresses changes in species richness providing valuable information on negative and positive changes separately. Then, species richness change (RC) was calculated using the difference in species number between pairs of years.

$$RC=\left(species\;number\;of\;plot\;in\;year\;2\right)-(species\;number\;of\;plot\;in\;year\;1)$$

RC was also averaged for each pair of year across patches, separately for burnt and unburnt patches.

These two types of distances were used as response variables against three explanatory variables. The first explanatory variable was the simple temporal distance, or time lag, that is, the time interval between the samples in a given pair of years. The second explanatory variable was the difference in precipitation in the growing seasons between the two years: precipitation of April to September of year two minus precipitation of April to September of year one. The third explanatory variable was the burning as a binary variable. The following models were built and tested:

- Dissimilarity (1-SM), as well as differences between dissimilarities in burnt and unburnt areas, vs. sampling time interval: Mantel test between 1-SM, or 1-SM in burnt area minus 1-SM in unburnt area, and time lag using Spearman correlation coefficient and 9999 number of unrestricted permutations. This was calculated separately for burnt and unburnt patches, as well as for the differences, and all pairs of years were involved in this analysis. These relationships were illustrated by fitting a linear regression line.
- Year-to-year species richness changes (RC), as well as the difference between burnt and unburnt areas, vs. precipitation change: linear mixed model of RC, on precipitation and burning as fixed factors and pairs of years as random factor. Only pairs of consecutive years were included.

All analyses were carried out for three sets of species. In the first case, all species were counted, 76 species in Orgovány and 62 species in Bugac. Then, long-lived (perennial herbs and woody species; 50 and 36 species, respectively) and short-lived (annual and biennial; 26 for both sites) species were differentiated and the analyses were carried out for each group. Calculations were performed using the R statistical software [36] using the ade4 package [37].

## Results

**Fig 1:**
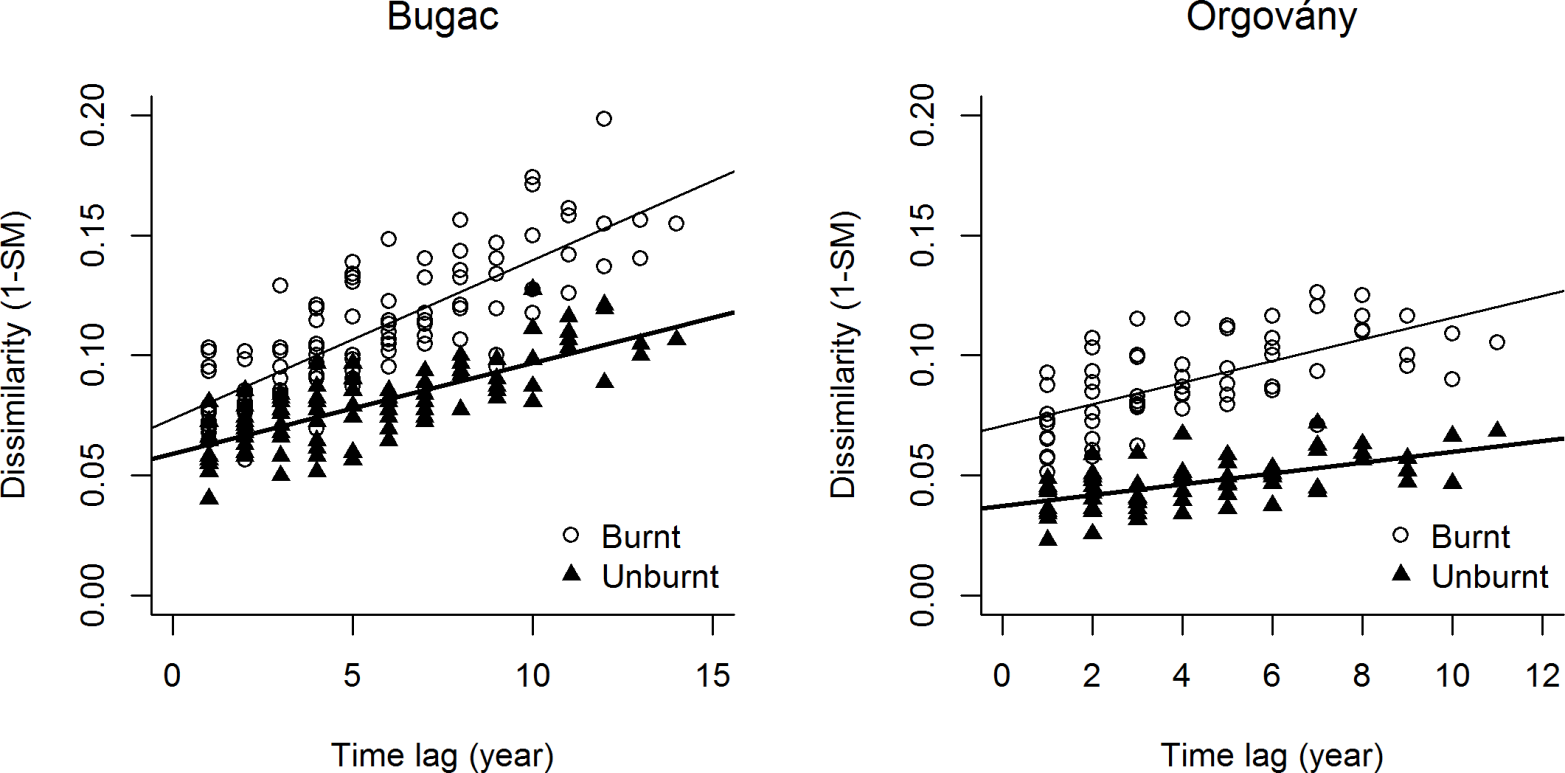
The compositional dissimilarity as a function of time lag on the two sites. Trend lines for illustrative purpose are fitted for significant relationships (bold for unburnt, simple line for burnt areas) according to Mantel-tests.

Compositional dissimilarity increased with the time intervals between the sampling events in both areas of both sites (Fig 1 and Section A of Table 2). The gradient of the increase was higher in burnt than in unburnt areas (see columns ‘Burnt-Unburnt’ of Section A of Table 2). Burnt areas were more variable and changed more over time than the unburnt areas—see Fig 1 for an example, where their dissimilarities are higher and have steeper gradients. We found the same results for the subsamples of the long-lived species. However, neither the composition of the short-lived plants, nor their burnt-unburnt differences changed significantly with the length of time intervals in the Orgovány site.

**Table 2:** Summary of statistical tests.

variables	site	Bugac			Orgovány		
	area	Burnt	Unburnt	Burnt-Unburnt	Burnt	Unburnt	Burnt-Unburnt
A) Dissimilarity vs time lag	All	***	***	***	***	***	**
	Long	***	***	***	***	***	***
	Short	**	**	+	+	-	+
B) Year-to year species richness change vs precipitation difference	All	***	**	**	***	*	**
	Long	-	-	-	*	*	-
	Short	***	**	**	***	*	***

“All”, “Long”, and “Short” refer to all species, long-lived and short-lived species subsamples, respectively. “Burnt-Unburnt” stand for the differences between the average dissimilarities (Section A) or burning and precipitation difference effects and their interactions on species richness difference (Section B). The signs show the significance of Mantel test (Section A) or the parameters of the linear mixed model (Section B):

*** p<0.001

** p<0.01

* p<0.05

+ p<0.1

- p>0.1

**Fig 2:**
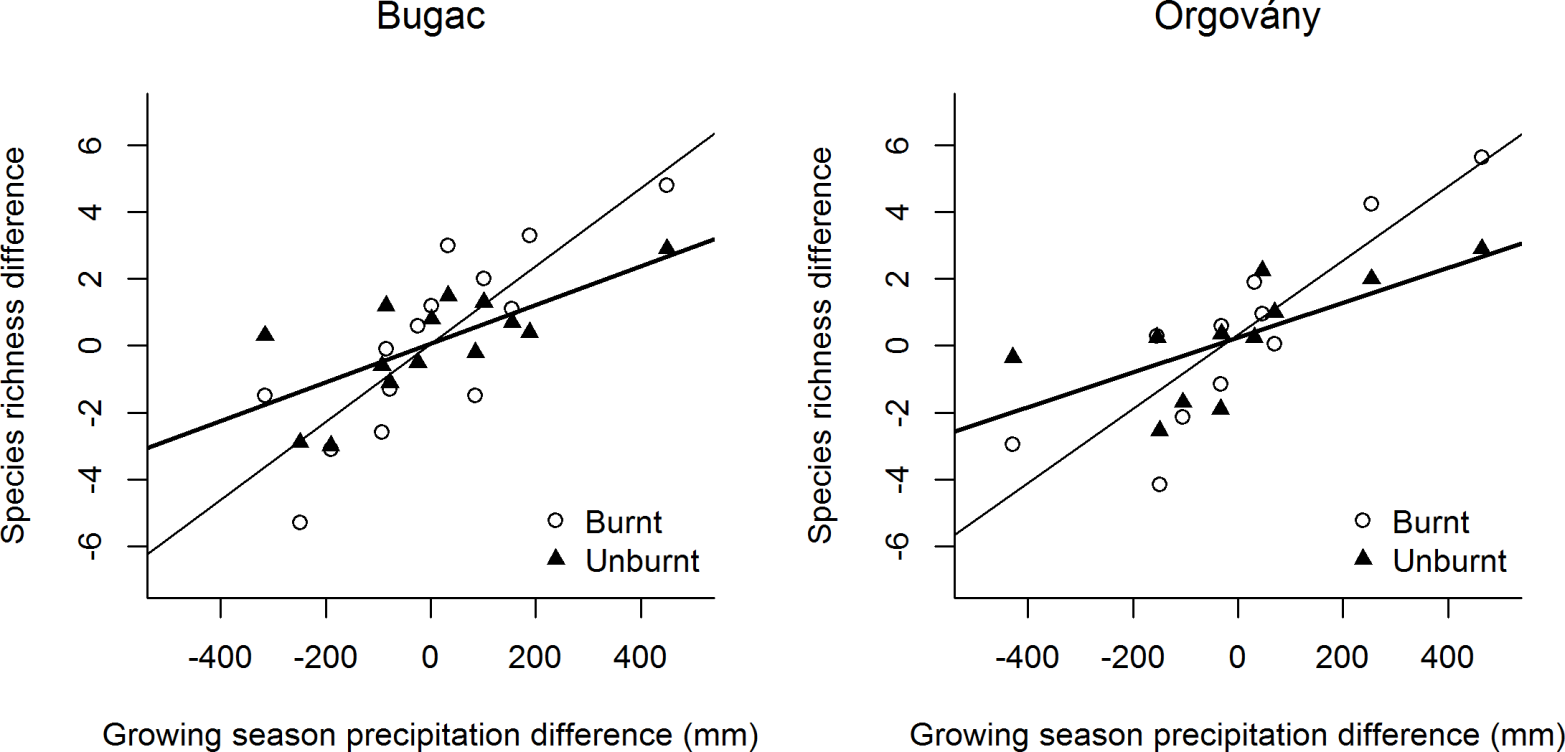
The relationship between year-to-year differences in growing season precipitation and species richness. Trend lines denote significant (p<0.05) regressions. Bold trend lines stand for unburnt areas, and regular ones for burnt areas.

We found that the species richness difference between consecutive years significantly increases with the precipitation difference of those years (Fig 2 and Section B of Table 2). In the case of burnt patches, the increase is significantly steeper compared to in unburnt patches (Section B of Table 2, Burnt-Unburnt columns). The subsamples of short-lived species show the same statistical relationship as the all-species samples. The richness of long-lived species changed with the year-to-year precipitation difference only in the Orgovány site, and we found no differences between the burnt and unburnt areas.

## Discussion

The two study sites provided similar results regarding year-to-year changes in species richness, as well as in terms of long-term compositional changes. In our previous study, we found that open sand grassland patches of low productivity regenerate quickly after wildfires [33], similar to the results of Knops [38], while the juniper trees, the major woody component in our sites, has not regenerated for decades [26]. We did not find post-fire juniper establishments during this study. This finding is in line with research done in the southwestern United States, where fire was recommended for the removal of juniper [39,40]. Even the poplar sprouts showed noticeably limited growth during the 12 or 15 years of the study, as none of them started to grow to tree in the sampling quadrats. Thus, due to the similarities in the results from the two sites we conclude that the major factor behind the differences of burnt and unburnt grassland dynamics was the long-term removal of the woody component by the wildfires, which determined grassland dynamics in the long run.

We found that all grassland areas that were studied changed significantly over a decadal time span (Fig 1). The burnt areas yielded greater long-term changes than the unburnt areas (Section A of Table 2, Burnt-Unburnt columns). This significant result in both study sites shows that the compositional dissimilarity grew faster in the burnt areas. We interpret this difference to mean that burnt grasslands proved to be more dynamic, which is in line with our previous result that there were more changes in the patch types of the grassland vegetation in the burnt areas [26].

The long-lived species consistently showed long-term dynamics, even in the unburnt areas, and the results for this set of species were the same as the results for all-species samples (Section A of Table 2, long-lived species). We found that the short-lived species showed long-term dynamics in the Bugac site (Section A of Table 2, short-lived species). In contrast, in Orgovány, the short-lived species did not show long-term dynamics. We assume that in this case, the short-lived species were permanently present in the seed bank and appeared and disappeared according to the moisture availability as a short-term environmental filtering agent [41].

Regarding our second question, species richness significantly changed with year-to-year differences in precipitation at each site (Fig 2). The three largest year-to-year precipitation differences since 1900 occurred after 1999, during our study period, in the nearby Kecskemét Meteorological Station of the Hungarian Meteorological Service (S1 Fig). The close relationship between year-to-year changes in species richness and year-to-year differences in growing season precipitation underlines the importance of this pattern. Our finding is similar to the results of Keeley et al. [42] who reported a consistently significant positive precipitation effect on species richness in a 90-site 5-year Californian shrubland study. However, we found that year-to-year differences in growing season precipitation caused significantly more changes in species richness on the bunt areas than on unburnt areas in both study sites (Section B of Table 2, Burnt-Unburnt columns). Although post-fire vegetation dynamics is well-studied, we did not find any research explicitly comparing the effect of inter-annual precipitation variability on species richness in burnt and unburnt areas. The stronger effect of year-to-year precipitation differences on the burnt vegetation is most likely due to the higher range of temperature and air humidity in the unshaded patches [43]. Indeed, plant biomass removal by fires or by other means may contribute to making those stands more open [19] and warmer [21], magnifying the effects of drought.

The short- and long-lived species contributed differently to the year-to-year dynamics. In the case of long-lived species, we found statistically significant year-to-year dynamics only in the Orgovány site (Section B of Table 2, long-lived species). This difference between Bugac and Orgovány might be the consequence of the different long-lived species pool; in Orgovány, some matrix species’ size can considerably change between years, and thus the species can appear in or disappear from the sampling units. The short-lived species strongly responded to the year-to-year changes in precipitation. This result is in accordance with the 29-year long observation of Yan et al. [44] in a dry steppe, who reported higher variability of annual biomass in more dry years and strong correlation between precipitation and annual species richness. Cleland et al. [13] found that among the grassland experiments of the US LTER programme, both relative abundance of annuals and year-to-year species turnover increased with aridity. These findings correspond to the “pulse” behavior of semi-arid or arid grassland annuals in a “pulse-reserve” paradigm [45] in which] the annuals are adapted to utilize shorter-term resources to growth and propagation, which in turn may contribute to a reserve in form of seed bank. We found higher changes of short-lived species richness in the burnt areas, which might be due to the more extreme microclimate [21,43], as discussed earlier. We interpret our finding to mean that the “pulse” behavior of the short-lived species is boosted by the more extreme microclimate of the burnt areas.

## Conclusion

We found that compositional changes in decadal time span and year-to-year changes of species richness are both affected by previous fire events. These interactive effects of burning and sampling time interval or year-to-year precipitation difference can be detected in two separate forest-steppe stands, one of which burnt decades before and the other two years before the beginning of the study. The most important decadal change of the two studied burnt areas is that the woody vegetation component was decreased by wildfires and only poplar species started a slow regeneration. Thus, land use change, and specifically afforestation by black pine, was found to induce indirect changes in the vegetation pattern and microclimate of grasslands in forest-steppe vegetation via more frequent and severe wildfires. Furthermore, climate change is expected to increase the incidence of droughts and heatwaves. Thus, these factors of global change, including woody canopy removal by wildfires and increasing inter-annual variability of precipitation, act in a synergistic way and result in greater long-term and year-to-year changes in the composition of the grasslands of forest-steppe. Our results draw attention to the protection of the woody element of the remaining forest-steppe stands from the effects of the land use change. The damage to the woody component makes the grassland component more sensitive to precipitation variability, accelerating the vegetation dynamics, thus may endanger reaching conservation goals.

## Supporting information

S1 Fig The year-to-year differences in precipitation between 1901 and 2015, Kecskemét meteorological station. The three highest year-to-year differences, i.e. 1999–2000, 2009–2010, and 2010–2011, as well as the annual minimum (2003) and maximum (2010) precipitation occurred during our study period of 1997–2013.
(TIF)

S1 Dataset Vegetation data from Bugac and Orgovány sites. Plant names are used according to The Plant List (Version 1.1. Published on the Internet. 2013. Available: http://www.theplantlist.org/).
(XLSX)

S2 Dataset Seasonal precipitation data from Bugac and Fülöpháza meteorological stations. Fülöpháza data were used for Orgovány site, 6 km from the meteorological station.
(XLSX)
