# Supplementary figures and images for: Synergistic effects of the components of global change: Increased vegetation dynamics in open, forest-steppe grasslands driven by wildfires and year-to-year precipitation differences
Source: PLoS One. 2017 Nov 17;12(11):e0188260. doi: 10.1371/journal.pone.0188260 (PMC5693438; doi:10.1371/journal.pone.0188260)

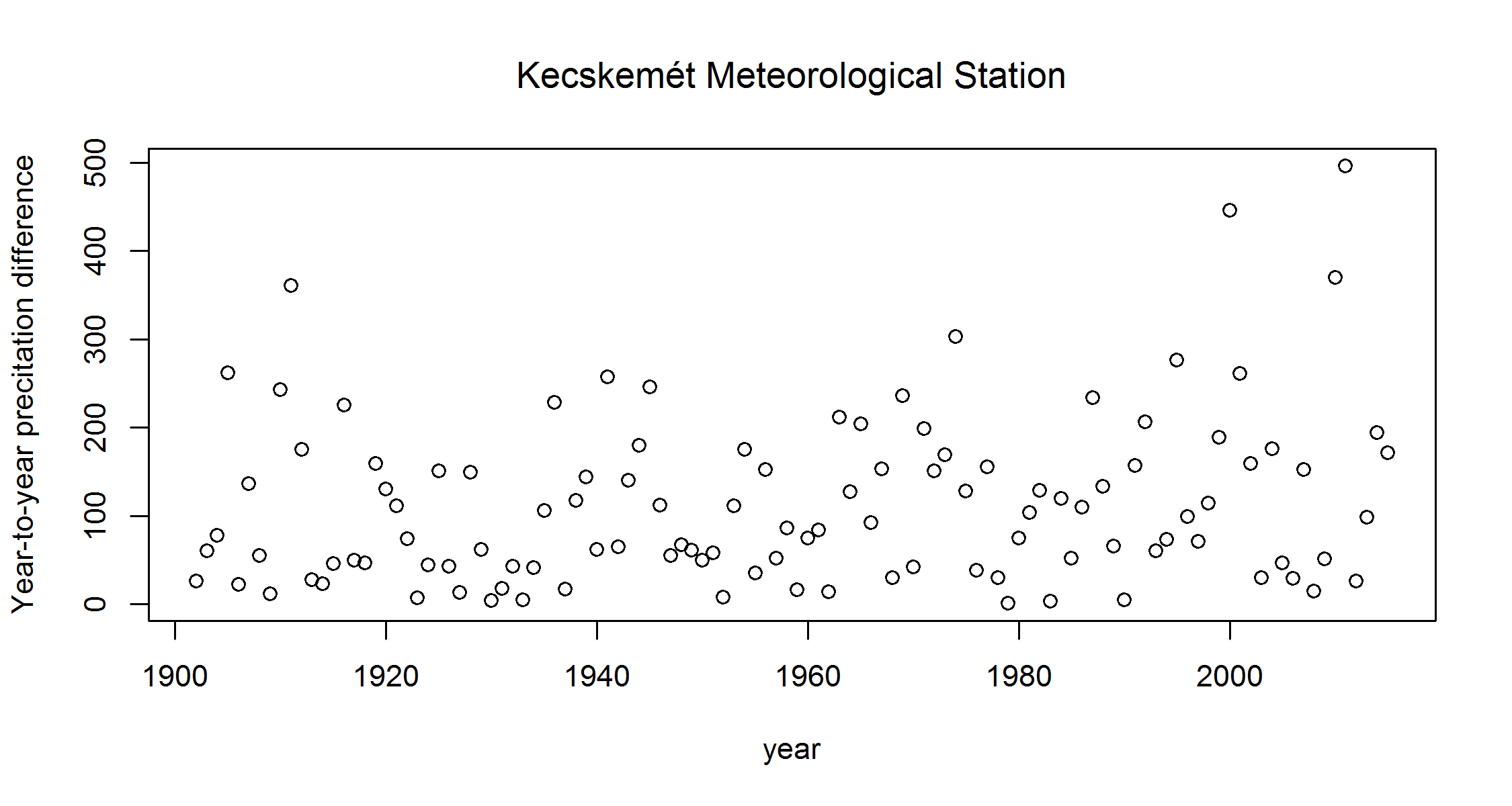

Supplement: S1 Fig — The three highest year-to-year differences, i.e. 1999–2000, 2009–2010, and 2010–2011, as well as the annual minimum (2003) and maximum (2010) precipitation occurred during our study period of 1997–2013. (TIF) [file pone.0188260.s001.tif]
